# Neoadjuvant Immuno-Chemotherapy: A New Perspective for Stage III NSCLC?

**DOI:** 10.3389/fsurg.2022.843987

**Published:** 2022-04-05

**Authors:** Yuanshan Yao, Dongfang Tang, Wen Gao, Huibiao Zhang

**Affiliations:** Shanghai Key Laboratory of Clinical Geriatric Medicine, Department of Thoracic Surgery, HuaDong Hospital Affiliated to Fudan University, Shanghai, China

**Keywords:** immuno-chemotherapy, stage III non-small cell lung cancer, surgery, NSCLC, ICI

## Abstract

**Background:**

Stage III Non-small cell lung cancer (NSCLC) is a heterogenous disease with novel treatment options. Recently, immunotherapy has attracted a lot of attention for advanced NSCLC.

**Objective:**

The objective of our study was to assess the efficacy and safety of neoadjuvant immuno-chemotherapy for resectable stage III NSCLC.

**Methods:**

We analyzed 11 stage III primary NSCLC surgical cases who had undergone standard lobectomy or bronchial sleeve resection and lymph node dissection between December 2020 and July 2021. The data analyzed included basic clinical features, serum levels of key biomarkers, clinical efficacy in the perioperative period, postoperative pathological results, postoperative complications and the incidence rates of Immune-Related Adverse Events.

**Results:**

Eleven patients were enrolled in our study with a mean age of 67.7 ± 4.8 years, and 10 patients being men with former or current smoking history. Squamous carcinoma (10/11, 91.1%) was the most common cancer type. Six patients had stage IIIa, five had stage IIIb. All patients received two or three cycles of neoadjuvant immuno-chemotherapy, with the median duration between the last treatment and surgery being 39 days (range, 32–46 days). All patients underwent R0 resection with ten patients undergoing single-port video-assisted thoracoscopic surgery. The median operative time was 170 min (range, 120–240 min). Only three (3/11, 27.3%) patients experienced mild postoperative complications and the mean hospital stay time was 6.9 days (range, 4–15 days). Nine (9/11, 81.8%) patients experienced major pathological response of which seven (7/11, 63.6%) was complete pathological response in postoperative results. The pathological stage was downgraded in 10 (10/11, 91.1%) patients, and although the incidence of Immune-Related Adverse Events was slightly higher (8/11, 72.7%), most events were grade 1–2 and did not delay surgery.

**Conclusion:**

Our study demonstrated that neoadjuvant immuno-chemotherapy is feasible and relatively safe for resectable stage III primary NSCLC patients. We hope this new neoadjuvant immuno-chemotherapy model can improve overall survival and open a new era for stage III primary NSCLC patients.

## Background

Stage III NSCLC is a heterogenous disease whose treatment options include surgery, molecular targeted therapy, chemotherapy, and radiotherapy. However, the 5 year overall survival is only about 15.8% (1). This outcome is unsatisfactory since the lung malignancies and metastatic lymph-nodes in stage III NSCLC are mainly confined in thoracic cavity and mediastinum or supraclavicular lymph node, unlike in stage IV NSCLC. Therefore, these patients are considered to have better prognosis and are expected to have higher overall survival rates (2). According to NCCN guidelines, patients with potentially resectable stage IIIA NSCLC should first receive two cycles of induction chemotherapy, followed by surgery for those who have not progressed after chest enhanced CT assessment. However, induction therapy has been shown to enhance 5 year overall survival rate by only 5% (3). Nevertheless, the development of immunotherapy has the potential to significantly enhance survival rate. The CheckMate 017 and CheckMate 057 phase III trials demonstrated that immunotherapy alone improved overall survival and was feasible for advanced NSCLC compared with docetaxel (4). In the NADIM phase 2 trial, neoadjuvant immuno-chemotherapy enhanced the 2-year overall survival rate in 41 Stage IIIa NACLC patients by 90% (5).

There is no denying that immunotherapy has revolutionized the landscape of advanced NSCLC. However, many thoracic surgeons fear that hyperprogression may occur in a subset of Stage III NSCLC patients and conversely accelerate tumor growth that can result in death. This novel tumor progression has been observed in 4–29% of patients (6). Meanwhile, the incidence of Immune-Related Adverse Events has been reported to be 30–70% in advanced stage lung cancer patients (7), with the rate of immunotherapy-induced interstitial pneumonitis being ~6% in NSCLC patients (8). Although interstitial pneumonitis is uncommon, it is the most serious complications of immunotherapy. Patients who were originally considered to be resectable and suffer interstitial pneumonitis may not be eligible for surgery after cure.

Therefore, there is need to evaluate the efficacy and safety of neoadjuvant immuno-chemotherapy for resectable stage III NSCLC. The aim of our study was to assess the efficacy and safety of neoadjuvant immuno-chemotherapy for resectable stage III NSCLC and establish a new therapeutic strategy.

## Methods

### General Materials

This was a retrospective study that enrolled Stage III primary NSCLC patients who had undergone neoadjuvant immuno-chemotherapy followed by surgery in HuaDong hospital from December, 2020 to July, 2021. Included criteria were as follows: (I) The patients had been diagnosed with non-small cell lung cancer before intervention using transthoracic lung aspiration or bronchoscopy biopsy, and classified as clinical Stage IIIa, IIIb, and IIIc based on the eighth edition of the TNM classification for lung cancer (9); (II) All enrolled patients underwent standard lobectomy or bronchial sleeve resection and lymph node dissection; (III) Patients underwent neoadjuvant chemo-immunotherapy after consultation between patients and thoracic surgeons and discussion by a multi- disciplinary team; (IV) Patients whose coagulation was normal. Exclusion criteria were as follows: (I) After two or three cycles of neoadjuvant immuno-chemotherapy, PET-CT displayed that elevated standard uptake value in N3 station lymph nodes; (II) Patients whose coagulation was abnormal; (III) Patients whose lung function was so poor that can not tolerate operation. The neoadjuvant chemo-immunotherapy regime was determined by pathological results. Patients with adenocarcinomas were put on pemetrexed plus carboplatin combined with PD1 inhibitor, while patients with squamous carcinoma were put on albumin-bound paclitaxel plus carboplatin combined with PD1 inhibitor. Routine laboratory blood test including blood gas analysis, whole body PET-CT, lung function and chest contrast-enhanced CT were performed before the surgery.

**Figure 1:**
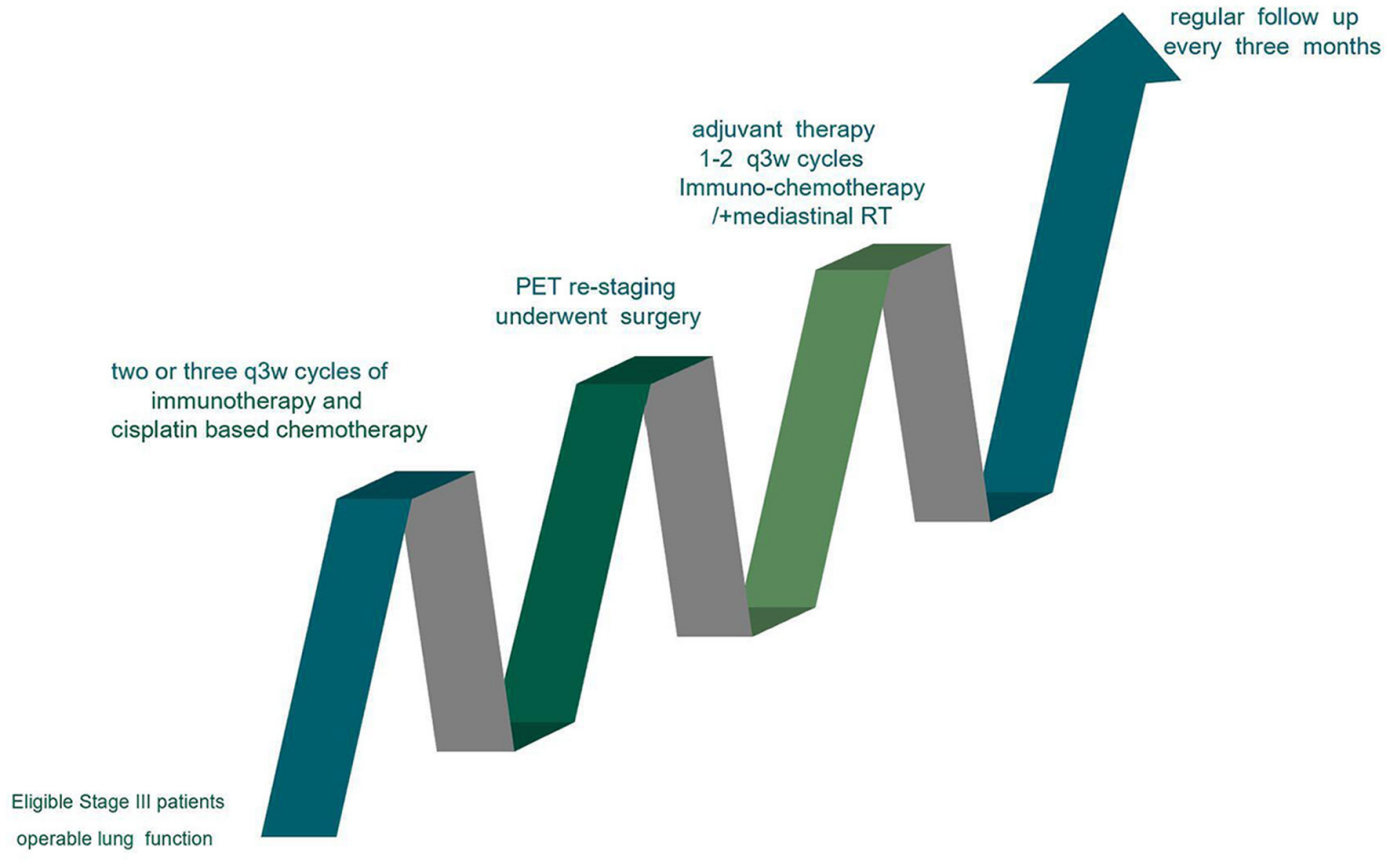
Standard procedure for the treatment of resectable stage III patients.

The standard procedure used for the treatment of resectable Stage III patients is shown in Figure 1. The CONSORT Flow Diagram was listed in Table S1.

The study was conducted in accordance with the Declaration of Helsinki (as revised in 2013). The study was approved by ethics board of Huadong Hospital and informed consent was obtained for each patient.

### Study Methods

Baseline clinical characteristics including sex, age, gene mutational status, and chemo-immunotherapy response were retrieved from the hospital records. We also retrieved data on pre- and post-treatment serum levels of circulating inflammatory biomarkers, such as C-reaction protein, NLR (neutrophil to lymphocyte ratio), PLR (platelet to lymphocyte ratio), and serum SCC (squamous cell carcinoma antigen) as well as immune related indexes including IL-1β, IL-6, and CD4/CD8. We also analyzed intraoperative and postoperative recovery efficacy using duration of the operation, intraoperative blood loss, pleural adhesion, time of chest tube removal, volume of thoracic drainage and average time of hospital stay. Postoperative pathological results, postoperative complications and incidences of Immune-Related Adverse Events were also recorded.

Immune-Related Adverse Events were recorded according to the Common Terminology Criteria Adverse Events developed by the National Cancer Institute (10). Radiologic response assessment was explored based on the irRecist criteria (11). Major postoperative pathological response was indicated by the presence of <10% active tumor cells in postoperative pathological specimens (12). Radiological and pathological responses were assessed by two experienced doctors, who resolved conflicting opinions through discussion. Continuous variables were expressed as mean ± standard, while categorical variables were expressed as numbers and percentages. Intragroup variances between pre- and post- intervention data were compared using paired sample *t*-test. *P* < 0.05 was considered significant.

## Results

### Clinical Demographics

A total of 11 patients who underwent immuno-chemotherapy followed by surgery were enrolled in this study between December, 2020 and July, 2021. The baseline clinical parameters are summarized in Table 1. Ten patients had history of smoking. Preoperative TNM staging was as follows: 6 patients with stage IIIa; 5 patients with stage IIIb. Six IIIa patients included one cT1cN2M0, three cT2aN2M0, and two cT2bN2M0. Five IIIb patients included two cT2aN3M0 and three cT4N2M0. Three patients with <1% PD-L1 expression were defined as PD-L1 negative, while 4 patients with more than 1% PD-L1 expression were defined as PD-L1 positive. The pre-immuno-chemotherapy PD-L1 expression data for four patients was lacking. Since no patients harbored EGFR or ALK gene mutation, none of the patients received targeted therapy. Average follow up time was 10.4 months. Up to the writing of this manuscript, none of the patients had relapsed and they all undergo regular follow up in our hospital.

**Table 1:** Clinical data of patients.

**General data**	
Age (years old)	67.7 ± 4.8
Gender (male/female)	10/1
Past history [*n* (%)]	
Smoking	10 (91.1)
Hypertension	7 (63.7)
Diabetes	3 (27.3)
COPD	7 (63.7)
Idiopathtic pulmonary fibrosis	1 (8.9)
Maximum diameter of lesion (cm) (range)	5.7 ± 1.4 (3.2–8.2)
Lesion site [*n* (%)]	
Left upper lobe	4 (36.3)
Left lower lobe	2 (18.2)
Right upper lobe	3 (27.3)
Right lower lobe	2 (18.2)
Pathological type [*n* (%)]	
adenocarcinoma	1 (8.9)
squamous carcinoma	10 (91.1)
Pathological stage [*n* (%)]	
IIIA	6 (54.6)
IIIB	5 (45.4)
PD-L1 expression level	
<1%	3 (27.3)
≥1%	4 (36.3)
unknown	4 (36.3)
FEV1%	
Mean ± SD (range)	77.2 ± 9.1 (55–95)
EGFR mutational status [*n* (%)]	0
ALK mutational status [*n* (%)]	0
Surgical method	
VATS	10 (91.1)
Thoracotomy	1 (8.9)

### Preoperative Treatment

Eleven patients underwent 2 or 3 cycles of immuno-chemotherapy where one patient received pemetrexed plus carboplatin combined with PD1/L1 inhibitor, and the other ten patients received albumin-bound paclitaxel plus carboplatin combined with PD1/L1 inhibitor. Camrelizumab (7/11, 63.6%) was the most commonly used PD1/L1 inhibitor, with the other 4 patients receiving Durvalumab. The median duration between the last treatment and surgery was 39 days (range, 32–46 days). Preoperative CT scans displayed complete response in 2 patients, partial response in 6 patients and stable disease in 3 patients. None of the patients showed progression or hyper-progression. The pre- and post-intervention serum levels of several biomarkers were compared using paired sample *t-*test and are shown in Table 2. Complete response, partial response and stable disease were all considered to be indicators of effective response to immuno-chemotherapy. The results showed that immunotherapy was beneficial for patients with decreased NLR, PLR, and IL-6 levels (Table 2).

**Table 2:** Comparison of changes in serum laboratory biomarkers of patients between before immuno-chemotherapy and after cycles of immuno-chemotherapy.

**Serum biomarkers**	**Before treatment**	**After treatment**	***P***
C-reaction protein (ng/mL)	9.9 ± 3.3	8.4 ± 1.2	0.764
NLR	6.9 ± 0.4	3.9 ± 0.2	0.025
PLR	197.2 ± 15.4	170.9 ± 12.4	0.022
SCC (ng/mL)	5.9 ± 1.3	5.5 ± 1.6	0.974
IL-1β (pg/mL)	3.9 ± 1.3	3.3 ± 1.7	0.542
IL-6 (pg/mL)	33.8 ± 3.3	17.9 ± 2.9	0.009
CD4/CD8	2.9 ± 0.3	3.1 ± 0.4	0.148

### Intraoperative Status

All patients underwent successful surgery. Ten patients underwent single-port video-assisted thoracoscopic surgery while one patient underwent a thoracotomy through traditional lateral posterior incision. The thoracotomy begun as a video-assisted thoracoscopic surgery, but was converted to open surgery because of extensive thoracic adhesions. Blood loss, average operation time, and pleural adhesions are shown in the Table 3. The surgeries took long because since all of the patients had extensive thoracic adhesions.

**Table 3:** Intraoperative situation of patients.

**Intraoperative situation**	**Median (range)**
Operative time (min)	170 (120–240)
Intraoperative blood loss (mL)	390 (200–750)
Pleural adhesion [*n* (%)]	11 (100%)

### Postoperative Information and Postoperative Complications

None of the patients underwent R1 or R2 resection. Total thoracic drainage volume, average hospital stay and chest tube removal time are shown in Table 4. One third of the patients experienced postoperative complications. Owing to adequate preoperative preparations and intraoperative care such as smoking cessation, inhalation therapy, correction of anemia, lung protective ventilation strategies and nutritional support, life-threating complications such as acute respiratory distress syndrome and bronchopleural fistula did not occur. Two patients experienced persistent air leakage for more than 5 days, which was completely stopped through intrapleural injection of hypertonic glucose solution. None of the patients experienced incision infection, thoracic hemorrhage or deep venous thrombosis. The postoperative complications observed are listed in Table 5.

**Table 4:** Postoperative recovery situation of patients.

**Postoperative indexes**	**Mean (range)**
Chest tube removal time (d)	5.4 (3–14)
Thoracic drainage volume (mL)	470 (240–1,320)
Average hospital stay time (d)	6.9 (4–15)
Visual analog scale (VAS) score	3.8 (2–6)

**Table 5:** Postoperative complications of patients.

**Postoperative complications (*n*/%)**	
Atrial fibrillation	1 (9.1%)
Pulmonary infection	1 (9.1%)
Pulmonary air leak	1 (9.1%)

### Postoperative Pathological Examinations

**Figure 2:**
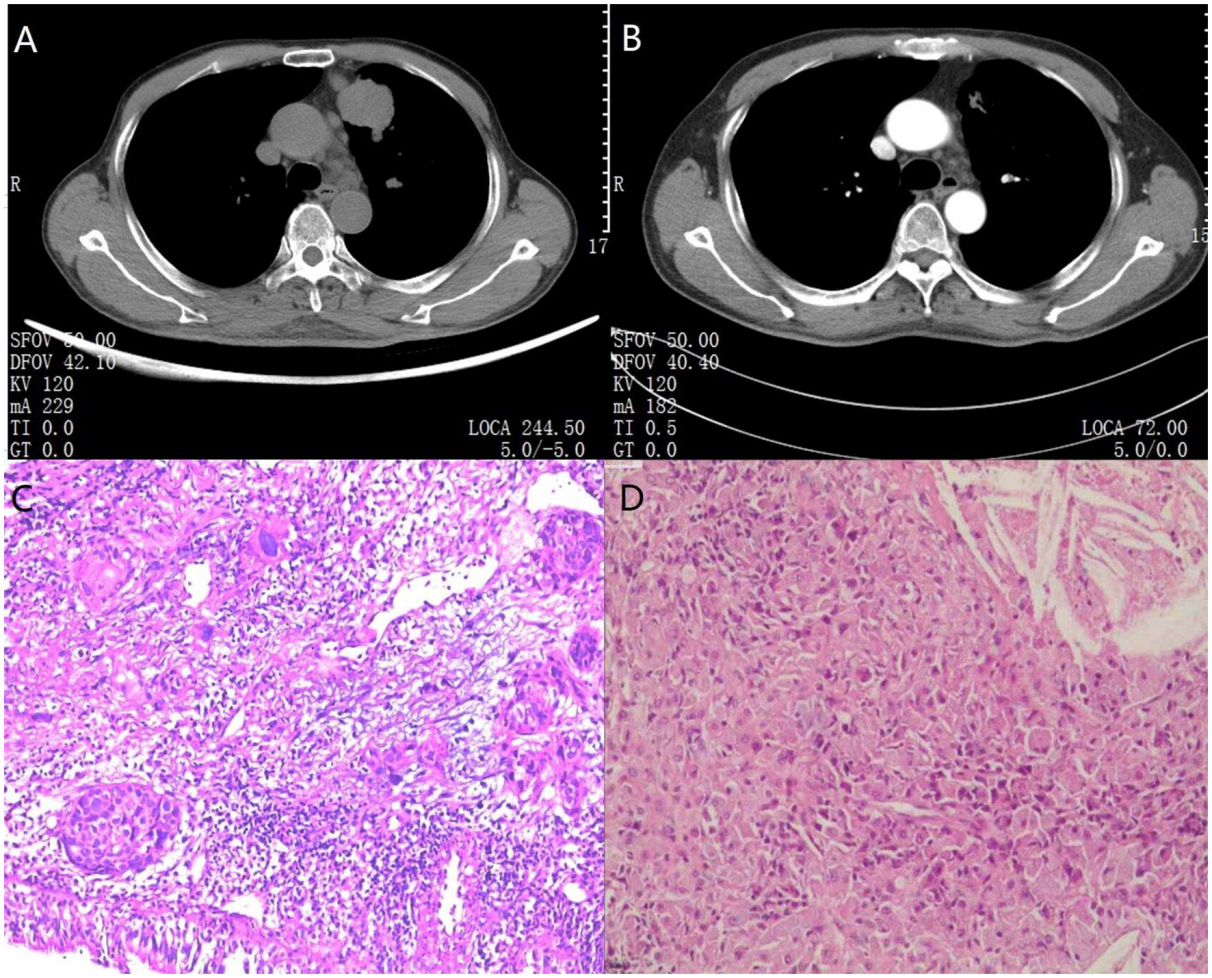
CT pre-treatment and after treatment combined with preoperative and postoperative specimens in one pathological completely response patient. **(A)** A 69-year-old man diagnosed with left upper lobe squamous carcinoma coupled with metastatic supraclavicular lymph nodes was classified as cT2aN3M0. Chest radiograph showed a large tumor (lung squamous carcinoma) located in the anterior segment of left upper lobe and enlarged aortopulmonary window lymph-nodes can be seen in the mediastinum. **(B)** After 2 cycles of immuno-chemotherapy, CT at the same level showed dramatic reduction in the size of the mass and number of enlarged lymph nodes. In addition, metastatic supraclavicular lymph nodes had vanished. After a left upper lobectomy and lymph node dissection, the patient recovered well and has not relapsed to date. **(C)** The results of percutaneous needle biopsy showed lung squamous carcinoma. **(D)** The postoperative pathological specimen indicated lack of residual lung cancer cells and lymph node metastasis. Only multinucleated giant cells and fibrotic tissue were observed. The magnification was 40 × for (C/D).

Postoperative pathological examinations demonstrated that 9 (81.8%) cases were major pathological response, of which 8 (72.7%) cases were pathological complete response. The pathological stage was downgraded in 10 (90.9%) patients after surgery. The Pre- and post-treatment CT scans as well as pre-and post-operation specimens of one random patient with complete pathological response are shown in Figure 2.

### Immune-Related Adverse Events During the Treatment Course

According to the Common Terminology Criteria Adverse Events developed by the National Cancer Institute, 8 (8/11, 72.7%) patients experienced grade 1–2 adverse effects mostly at 3–6 weeks after commencement of treatment (Table 6). No grade 3 or 4, abnormal laboratory results and life threatening adverse effects were recorded. None of the patients discontinued the treatment due to adverse effects. The most common adverse effect was grade 1–2 cutaneous rash, which resolved spontaneously without taking any medications (13). Four patients experienced grade 1–2 reactive cutaneous capillary endothelial proliferation during treatment with Camrelizumab, which quickly disappeared after a short time. Liver malfunction and anemia were the common abnormal laboratory results observed, which were solved by treating the symptoms.

**Table 6:** Immune related adverse effect.

	**Grade I**	**Grade II**
Cutaneous rash	5 (45.5%)	1 (9.1%)
Fatigue	3 (27.3%)	0
Nausea	1 (9.1%)	0
Reactive cutaneouscapillary endothelial proliferation	3 (27.3%)	1 (9.1%)
Anemia	1 (9.1%)	1 (9.1%)
Liver malfunction	2 (18.2%)	0
Pruritus	1 (9.1%)	0

## Discussion

Lung cancer is one of the most common malignancies worldwide. The role of surgery, the mainstay in the management of lung cancer, in NSCLC is simple and clear according to guidelines. The guidelines recommend curative surgery alone for stage I NSCLC patients and surgery followed by adjuvant chemotherapy for stage II patients. Surgery has also been reported to produce better outcomes for oligometastatic Stage IV NSCLC patients (14, 15). Novoa et al. showed that adrenal and lung oligometastatic patients who underwent curative surgery had a longer median overall survival compared with those who did not undergo surgery (14). However, the role of surgery in Stage III NSCLC presents a serious therapeutic dilemma. According to the 8th edition of TNM Classification for NSCLC, Stage III NSCLC is a heterogenous disease divided into three types: IIIa, IIIb, and IIIc. Hence, the role of surgery for Stage III NSCLC needs to be explored further due to the different TNM subtypes. Although Stage III and IV are classified as late stage lung cancers, Stage III patients are eligible for surgical intervention even with pathologic lymph nodal status N3 unlike Stage IV patients (16). Although Stage III patients might be viewed as a localized lung cancer, other therapeutic approaches including chemotherapy and radiotherapy are still suboptimal (17).

Recently, Immunotherapy has altered the treatment landscape of late stage NSCLC patients and brought a new perspective to the application of surgery in Stage III patients. Immunotherapy alone or combined with chemotherapy has been shown to improve the overall survival of unresectable NSCLC patients (18). Therefore, as immunotherapy is being used in advanced NSCLC patients, neoadjuvant Immuno-chemotherapy is being evaluated in Stage III patients. Major pathological response after neoadjuvant chemotherapy was validated to imply better outcome in resectable NSCLC patients (19). Roman et al. analyzed 41 NSCLC patients who underwent 2–4 cycles of Immuno-chemotherapy and observed major pathological response in 82.9% of the patients (20). This is the largest study on immuno-chemotherapy published in PUBMED. Another trial (NCT02716038) demonstrated that 17 (57%) patients among 30 resectable stage IB–IIIA patients experienced major pathological response after treatment with intravenous atezolizumab combined with carboplatin (21). Pembrolizumab monotherapy alone also showed high rates of major pathological response and downgrade of pathological stage in a study by Jiang et al. The rate of major pathological response in our study was almost 40%, an encouraging result for thoracic doctors (22).

Three factors are involved in the definition of resectable Stage III patients. First, Stage III NSCLC includes IIIa, IIIb, and IIIc according to the 8th edition TNM of lung cancer, while N3 station lymph node is stratified into Stage IIIb-c. N3 station lymph nodes mainly comprise supraclavicular and contralateral mediastinal lymph nodes. In preoperative assessments, PET-CT should be performed to confirm lack of apparent metabolism or proliferation in these N3 station lymph nodes. The use of contrast-enhanced CT techniques assist to detect such abnormal N3 station lymph nodes through the evaluation of N3 station lymph nodes enhancement patterns. Eligible of N3 patients who underwent surgery was cautious. N3 patients can accept surgical option only if the above two requirements were met. Secondly, mediastinal lymph node involvement may be an independent risk factor for 5-year survival of lung cancer patients (23). Preoperative bulky N2 disease after induction therapy was not a potential candidate for surgery (24). Thirdly, in clinical practice, several patients classified as Stage III after PET-CT or enhanced CT assessment decline induction therapy followed by surgery, and opt for surgical exploration alone. During surgery for these patients, intraoperation pathology results showed lack of metastasis in these clinically positive lymph nodes leading to downgrading of the pathological stage. Retrospective analysis showed that these patients might have lost an opportunity to undergo surgery if CT scans had demonstrated stable disease after induction therapy. So, Patients with stage III lung cancer should not preclude the chance of surgery easily.

In our study, although only 11 patients were enrolled, we demonstrated that neoadjuvant immuno-chemotherapy for resectable stage III NSCLC was safe and feasible. Most of the patients in our study had squamous carcinoma without gene mutations. After 2–3 cycles of immuno-chemotherapy, nine (9/11, 81.8%) cases experienced major pathological response, of which seven (8/11, 72.7%) cases were pCR. In addition, the pathological stage was downgraded in 10 (90.9%) patients. These results are similar to results of the NADIM trial (5). A recent study has demonstrated that pemetrexed chemotherapy can induce death of tumor cells, accelerate activation of CD8 T cells and induce upregulation of target antigens, thus altering the tumor microenvironment and increasing the efficacy of immunotherapy (25). Gemcitabine has also been shown to reduce myeloid suppressor cells and increase CD8T and NK56 anti-tumor cells in an animal model (26). These findings suggest that induction immuno-chemotherapy has therapeutic potential.

We observed a high rate of major pathological response, despite preoperative CT scans demonstrating that three patients had stable disease according to the irRecist standard. These results indicated that some patients might experience better outcomes from immunotherapy without findings of tumor shrinkage from thoracic imaging and the possible reason is immune cells infiltration, rather than tumor progression (27). Hence, the irRecist criteria is not always an optimal method to assess the outcomes of immunotherapy. Moreover, even some tumors were observed in an increase of enhanced CT may not preclude the chance of surgery.

Currently, PD-L1 expression in tumor cells, tumor-infiltrating lymphocytes and tumor mutational burden have been reported to be closely associated with the efficacy of immunotherapy (28). In our study, we also evaluated the predictive value of serum inflammatory biomarkers for immunotherapy. Recently, some studies showed that pre-treatment levels of NLR and PLR had prognostic value for NSCLC patients undergoing immunotherapy (29–31). Our results indicated that patients with significantly decreased NLR, PLR, and IL-6 levels may benefit from immunotherapy. We postulated that decreased NLR and PLR denoted an increase in lymphocyte cells. Tumor infiltrating lymphocytes are closely associated with the response to immunotherapy (32). IL-6 induces a broad range of acute phase and immune response proteins. Keegan et al. demonstrated that a decrease in IL6 level was related to better outcome of NSCLC patients (33). Recently, IL6 blockade was shown to exert anti-tumor effects in tumor bearing mice (34). In the era of combination treatments, combining anti-IL6 treatment with PD-1/PD-L1 inhibitors may provide an optimal approach for immunotherapy. In conclusion, these serum biomarkers might have prognostic value for immunotherapy.

All of these patients underwent successful surgery without any delay due to immuno-chemotherapy. After adequate preoperative preparations including strict cessation of smoking and respiratory training, the postoperative periods were uneventful with three patients encountering regular postoperative complications which would be solved through medications or just observation. The patients were also discharged from hospital after average hospital stay. Ten patients underwent single-port video-assisted thoracoscopic surgery and only one patient underwent conventional lateral posterior incision because of extensive thoracic adhesions. This patient underwent sleeve lobectomy, which was a challenging procedure due to thoracic adhesions and tighter lymph nodes around the bronchus. In summary, dense thoracic adhesions, tissue fibrosis and edema coupled with enlargement N1 or N2 lymph nodes increase intraoperative difficulty and blood loss. However, experienced thoracic surgeons can overcome this difficulty without increasing surgical complexity. In our cohort, there was one patient with idiopathic pulmonary fibrosis, a type of interstitial lung disease which can develop from acute exacerbation caused by surgery or immunotherapy (35, 36). Acute exacerbation of interstitial lung disease is a fatal disease that is almost incurable. In order to enhance the safety of this patient who also underwent lobectomy, we adopted prophylactic measures including high dose corticosteroid injection for 3 days, intraoperative low tidal volume ventilation, postoperative physiotherapy rehabilitation and intensive nutritional support which ultimately led to timely discharge from hospital (37).

Most thoracic surgeons fear the occurrence of Grade 3–4 immune related adverse events and thus do not recommend neoadjuvant immuno-chemotherapy. The irAEs are caused by immunotherapy mediated activation of immune system and can occur in many organs of the body (7). Majority of irAEs are mild and low-grade including rash, fatigue, nausea and liver malfunction. However, immunotherapy induced pneumonitis is fatal and results in 30–40% immunotherapy associated death (38). It is reported that the incidence rate of life threatening irAEs is low, and clinicians must pay more attention for the early identification and treatment of ICI-induced toxicity (7). It is also necessary to establish a nomogram prediction model to screen patients with a tendency to develop fatal adverse events. In our study, no grade 3–4 adverse effects were observed and all patients underwent surgery without any delay.

This study has some limitations. First, this was a retrospective study and only enrolled patients who had already undergone surgery. However, the proportion of patients with poor outcomes after neoadjuvant immuno-chemotherapy might be larger. Secondly, long term efficacy and survival rate of neoadjuvant immuno-chemotherapy remains unknown. Thirdly, the number involved in our study was small.

## Conclusion

In summary, our study showed that neoadjuvant immuno-chemotherapy is feasible and relatively safe for resectable stage III primary NSCLC patients. We hope that this new neoadjuvant immuno-chemotherapy model can improve overall survival and open a new era for stage III primary NSCLC patients. However, large scale cohorts are needed to verify the results.

## Data Availability Statement

The original contributions presented in the study are included in the article/Supplementary Material, further inquiries can be directed to the corresponding authors.

## Ethics Statement

The studies involving human participants were reviewed and approved by the study was conducted in accordance with the Declaration of Helsinki (as revised in 2013). The study was approved by ethics board of Huadong Hospital (No. 2021K094), and individual consent for this retrospective analysis was obtained. The patients/participants provided their written informed consent to participate in this study. Written informed consent was obtained from the individual(s) for the publication of any potentially identifiable images or data included in this article. Written informed consent was obtained from the patient for publication of this case report.

## Author Contributions

YY: conception and design, collection, and assembly of data. HZ and WG: administrative support. HZ: provision of study materials or patients. DT: data analysis and interpretation. All authors: manuscript writing and final approval of manuscript.

## Funding

This study was supported by the Shanghai Key Laboratory of Clinical Geriatric Medicine, Shanghai Municipal Key Clinical Specialty (shslczdzk02801), Digital technologies for the postoperative remote care and rehabilitation of thoracic and cardiac surgery patients (2019YFE0105600), and Diagnosis and Treatment Center of Lung cancer (H1382).

## Conflict of Interest

The authors declare that the research was conducted in the absence of any commercial or financial relationships that could be construed as a potential conflict of interest.

## Publisher's Note

All claims expressed in this article are solely those of the authors and do not necessarily represent those of their affiliated organizations, or those of the publisher, the editors and the reviewers. Any product that may be evaluated in this article, or claim that may be made by its manufacturer, is not guaranteed or endorsed by the publisher.
