# Supplementary material for: Neoadjuvant Immuno-Chemotherapy: A New Perspective for Stage III NSCLC?
Source: Front Surg. 2022 Apr 5;9:843987. doi: 10.3389/fsurg.2022.843987 (PMC9018103; doi:10.3389/fsurg.2022.843987)
Supplement: Supplementary file 1 [file Table_1.doc]

**CONSORT 2010 Flow Diagram**

**Allocation**

**Analysis**

**Follow-Up**

**Enrollment**

Assessed for eligibility (n=15 )

Excluded (n= 4 )

♦  Not meeting inclusion criteria (n=2 )

♦  Declined to participate (n= 1 )

♦  Other reasons (n= 1 )

Lost to follow-up (n= 0 )

Discontinued intervention (n= 0 )

Allocated to intervention (n= 11 )

♦ Received allocated intervention (n= 11 )

♦ Did not receive allocated intervention (give reasons) (n= 0 )

Analysed (n= 11 )
♦ Excluded from analysis (n=0 )

Randomized (n= 11 )
